# Effect of Stable Chlorine Dioxide and Vacuum-Packing Treatments on the Physicochemical and Volatile Flavor Properties of Pike Eel (*Muraenesox cinereus*) during Chilled Storage

**DOI:** 10.3390/foods11172701

**Published:** 2022-09-05

**Authors:** Qi Du, Xiaonan Chen, Huili Jiang, Bin Zhang

**Affiliations:** 1 Key Laboratory of Health Risk Factors for Seafood of Zhejiang Province, College of Food Science and Pharmacy, Zhejiang Ocean University, Zhoushan 316022, China; 2 Pisa Marine Graduate School, Zhejiang Ocean University, Zhoushan 316022, China

**Keywords:** pike eel, vacuum packaging, stable chlorine dioxide, muscle proteins, chilled storage

## Abstract

The effects of vacuum-packaging and stable chlorine dioxide treatments on the quality of pike-eel fillets were investigated during chilled storage for a period of up to 10 days. The results reveal that the sensory scores, total volatile basic nitrogen (TVB-N) content, total viable count (TVC), malondialdehyde (MDA) content, and the myofibrillar protein (MP) content of pike-eel fillets with different packing treatments all decreased significantly over 10 days of storage. However, the vacuum-packaging and stable chlorine dioxide pretreatment showed positive effects on the protein stability of pike-eel samples. Compared with the simple packaging (SP) and vacuum packing (VP) treatments, the combination treatments (CP) significantly inhibited the rapid increases in the TVB-N content, TVC values, and MDA content. Moreover, the comparative stability in the MP and its carbonyl content were maintained. Furthermore, our volatile organic compounds (VOCs) analysis confirmed that the combined packaging treatments significantly hindered protein and lipid oxidation, inhibited the growth of spoilage bacteria, and maintained the volatile flavors of pike-eel samples during chilled storage.

## 1. Introduction

The pike eel (*Muraenesox cinereus*), one of the most important demersal fish resources, is widely distributed in the Indian Ocean and Pacific Northwest. China has become the most important country for the export of pike-eel products in the world. Large amounts of pike eel are also consumed in China, Japan, and Korea, due to its unique taste and high nutritional value. Fresh pike eel is highly perishable after death due to the biochemical reactions caused by the growth and reproduction of microorganisms and the high abundance of endogenous enzymes in the muscle tissues [1]. Furthermore, pike eel is rich in proteins and lipids, which are easily utilized by bacteria. Thus, deterioration commonly occurs in pike-eel products during storage and transport. At present, pike eel and its products are usually preserved under frozen conditions. The products are also processed by a variety of methods, including pickling, deep-frying, and semi-drying [2,3]. However, it should be noted that the quality deterioration of pike-eel products still occurs during cold-temperature storage, which is primarily induced by the denaturation and oxidation of muscle proteins and lipids, especially during prolonged chilled or frozen storage. Therefore, studies on the flavor, texture properties, protein, and lipid content of pike eel and its products are significant to the pike-eel processing industry.

Vacuum packing is a preferred processing method that effectively maintains the color, microbial load, texture, and nutritional values of muscle [2,4]. Vacuum provides an anaerobic environment with good barrier properties toward air and water, inhibiting the growth of aerobic spoilage microorganisms, e.g., *Pseudomonas* spp., and limiting oxidative rancidity. Chan et al. reported that vacuum-packing treatments slowed microbial growth and extended the shelf-life of silver carp fillets during chilled storage [5]. Fidalgo et al. found that the physicochemical properties of Atlantic salmon muscle were significantly maintained by vacuum-packaging treatments during 30 days of storage at 10 °C [6]. However, there is concern about the survival and growth of both microaerophilic and anaerobic psychrotrophic pathogens. Additional techniques should be performed to ensure the safety and quality of vacuum-packaged products. Additionally, several additives, including chlorine dioxide, are widely used to control the growth of pathogenic organisms in fish, shrimp, and their products. However, chlorine dioxide gas has some safety concerns. When the concentrations are higher than those typically used for sterilization (>10%), chlorine dioxide gas is very active and even becomes explosive. Therefore, stable chlorine dioxide (steady-state, with high safety and efficiency) has been widely used in the field of aquatic products and vegetables in recent years. Stable chlorine dioxide is a solid precursor composed of chlorine dioxide and chlorous acid, and its solution is colorless with a slight chlorine odor [7]. Stable chlorine dioxide has been used to investigate the combined effects of erthorbate and gellan gum glazing on the quality of commercial peeled shrimp during frozen storage [8]. According to the national standard of China (GB 2760, 2014), stable chlorine dioxide is suggested for use in aquatic products (only fish processing) with a maximum dose of 0.05 g/kg muscle.

At present, there are few reports on the preservation effects of stable chlorine dioxide combined with vacuum-packaging treatments on fish and its products. The objective of the current study was to investigate the potential effects of stable chlorine dioxide pre-soaking and vacuum-packing treatments on the muscle quality of pike-eel fillets during chilled storage (at 4 °C) to develop an effective method for the preservation of pike-eel products.

## 2. Materials and Methods

### 2.1. Chemical Reagents

Stable chlorine dioxide was purchased from Shandong Huashi Pharmaceutical Co., Ltd. (Weifang, China). A malondialdehyde determination kit was obtained from Nanjing Jiancheng Bioengineering Research Institute (Nanjing, China). Trichloroacetic acid (TCA), boric acid, 2,4-dinitrophenyl hydrazine (DNPH), magnesium oxide (MgO), ethyl acetate, tris(hydroxymethyl)aminomethane (Tris), and guanidine hydrochloride were purchased from Sinopharm Group Chemical Reagent Co., Ltd. (Shanghai, China).

### 2.2. Fish Samples and Treatments

Fresh pike eels (*M. cinereus*) varying from 1.2 kg to 1.5 kg in weight were purchased from a local aquatic market in Zhoushan, China. Fish samples were placed in a box filled with crushed ice and then transported to the laboratory within 20 min. Upon arrival, they were taken out and washed with ice water. Subsequently, the fish samples were manually prepared by removing the head and visceral mass. After thoroughly washing with distilled water, the fish samples were sectioned into approximately 30 g (6 × 6 × 2 cm) pieces. After draining for 10 min, the obtained fish pieces were randomly divided into the following groups: SP group (samples soaked in sterile water for 10 min, drained for 10 min, and then packaged in polyethylene bags (15 cm × 10 cm × 0.19 mm, Beiguo Packing Equipment Co., Ltd., Wuxi, Jiangsu, China); VP group (samples soaked in sterile water for 10 min, drained for 10 min, and then vacuum-packed at −0.02 MPa (ZKT-450 SK, Zhejiang Ruibao Packaging Machinery Co., Ltd., Wenzhou, Zhejiang, China) in polyethylene bags); and CP group (samples soaked in 0.05% stable chlorine dioxide solution for 10 min, drained for 10 min, and then vacuum-packed −0.02 MPa in polyethylene bags). Finally, the packed samples were refrigerated at 4 °C for 10 days. Samples were removed at 2-day intervals (0, 2, 4, 6, 8, and 10 days) and further analyzed.

### 2.3. Sensory Analysis

Pike-eel samples were subjected to sensory analysis according to the method reported by Fan et al., using a nine-point hedonic scale method (1 = extremely dislike, and 9 = extremely like) [9]. The middle part of the fillets was cut into 2 × 2 × 2 cm cubes, and the sensory evaluations were performed immediately after removing the samples from their storage bags. Participants were asked to evaluate each blind-labeled sample once in the same clean independent sensory lab randomly. The rating panel consisted of seven trained laboratory personnel who assessed the color, odor, texture, histomorphology, and the overall acceptability of the pike-eel muscle samples.

### 2.4. Total Volatile Basic Nitrogen (TVB-N) Content Analysis

The TVB-N content of the pike-eel samples was determined during storage, according to the method of Cai et al., with minor modifications [10]. Ten grams of sample was homogenized with 75 mL distilled water in a digestion tube. Next, 1 g of MgO powder was added to the mixture, which was immediately loaded into an automatic Kjeldahl apparatus ((KDN-520, Shanghai Bangyi Precision Measuring Instrument Co., LTD, Shanghai China). The distillation was collected with a recipient flask containing 15 mL of 2% (*w*/*v*) boric acid solution and was titrated by using 0.01 mol/L hydrochloric acid solution. The TVB-N content was calculated according to hydrochloric acid consumption and expressed as mg/100 g protein:

(1) $$X=\frac{\;V\times c\times 14}{m}\times 100$$

where *X* indicates TVB-N content, *V* indicates hydrochloric acid consumption, and *m* indicates the weights of pike-eel samples.

### 2.5. Microbiological Analysis

Total viable count (TVC) determinations were carried out according to a modified method by Binsi et al. [11]. The samples (25 g) were homogenized in 225 mL of 0.85% sterile saline solution for 2 min. After dilutions, 1 mL of each dilution was transferred to a plate count agar (PCA; Qingdao Haibo Biotechnology Co., Ltd., Qingdao, China). The resulting plates were aerobically incubated at 30 °C for 72 h, and the TVC values were calculated by counting the number of colony-forming units on the plates.

### 2.6. Myofibrillar Protein (MP) and Carbonyl Content Analysis

Myofibrillar protein (MP) extracted from pike-eel muscle was prepared according to the method reported by Sun et al. [12]. The samples were minced and homogenized in 5 volumes of Tris-HCl buffer (10 mmol/L, pH 7.2) for 1 min. The resulting homogenate was centrifuged at 6000× *g* for 10 min at 4 °C, and the supernatant was removed. The extraction was repeated, and the combined sediment was mixed with 5 volumes of Tris-HCl buffer solutions (containing 0.6 mol/L NaCl) and homogenized again. The homogenate was extracted at 4 °C for 1 h and then centrifuged at 10,000× *g* for 15 min at 4 °C. The obtained supernatant was confirmed to be the extracted MPs. The concentration of MP was determined via the classical Lowry method with bovine serum albumin as a standard.

The carbonyl content of MPs was determined by following the procedures described by Xia et al., with slight modifications [13]. Briefly, the MP extracts were mixed with 10 mmol/L DNPH and incubated for 1 h in the dark. Then 20% (*w*/*v*) TCA solution was added to the mixture and centrifuged at 10,000× *g* for 15 min at 4 °C. The obtained precipitates were washed three times with an ethanol:ethyl acetate (1:1, *v*/*v*) solution. The final precipitates were dissolved in 20 mmol/L sodium phosphate buffer solutions (containing 6 mol/L guanidine hydrochloride) and incubated for 15 min at 37 °C. After centrifugation (10,000× *g*, 10 min), the absorbance of the supernatant was measured at 370 nm, using a U-2600 UV–Vis spectrophotometer (Shanghai Lonicke Instrument Co., LTD., Shanghai, China). The carbonyl content was expressed as nmol of DNPH fixed per mg of MPs:

(2) $$Y\;=\frac{A_{370}\times \;D}{\varepsilon \;\times \;C}$$

where Y indicates the carbonyl content, A_370_ indicates that the absorbance of the supernatant was measured at 370 nm, D indicates the dilution multiple, ε indicates the molar absorbency index, and C indicates the content of MPs.

### 2.7. Malondialdehyde Content Analysis

MDA content was determined by using an MDA assay kit (Nanjing Jiancheng Bioengineering Institute, Nanjing, China). Briefly, the samples of pike-eel muscle were homogenized in 9 volumes of saline solution, followed by centrifugation at 3000× *g* for 10 min at 4 °C. The supernatant was obtained and determined according to the kit’s operating instructions. The absorbance values were measured at 532 nm, using a U-2600 UV–Vis spectrophotometer.

### 2.8. Volatile Compound Analysis

Gas chromatography (GC) coupled with a high-resolution ion mobility spectrometry (IMS) (FlavourSpec^®^, Hanon Advanced Technology Group Co., Ltd., Jinan, China) was used to determine the volatile compounds of the samples during storage (0, 4, and 8 days), according to the method proposed by Chen et al. [14]. Two grams of fish muscle was placed in a 20 mL headspace vial. After incubation for 20 min at 40 °C, 500 µL of headspace gas was automatically drawn by a syringe needle (85 °C). The pre-separation was performed on an FS-SE-54-CB-1 capillary column (15 m × 0.53 mm) at 60 °C. Nitrogen gas was employed as a carrier gas with the following program: an initial flow rate of 2 mL/min for 2 min, which was increased to 10 mL/min for 8 min, then increased to 100 mL/min for 10 min, and was finally maintained at 150 mL/min for 5 min. The total run time was 25 min. The elution was performed in isothermal mode, and the analytes were ionized in the IMS ionization chamber (temperature, 45 °C; flow rate, 150 mL/min). The retention index (RI) of volatile organic compounds (VOCs) was calculated with reference to n-ketone C4–C9 (Sinopharm Chemical Reagent Beijing Co., Ltd., Beijing, China). VOCs were identified in the GC–IMS data library based on the drift time and RI comparison. The signal intensity was expressed by peak height or peak area.

### 2.9. Data Analysis

The results are the average of three repeated experiments, except where otherwise specified. Data are expressed as the mean values ± standard errors and were analyzed via statistical analysis software (SPSS Inc., Chicago, IL, USA). Analysis of variance was performed between means to determine the significant differences by using Duncan’s multiple range test (*p* < 0.05).

## 3. Results

### 3.1. Sensory Property Analysis

**Figure 1:**
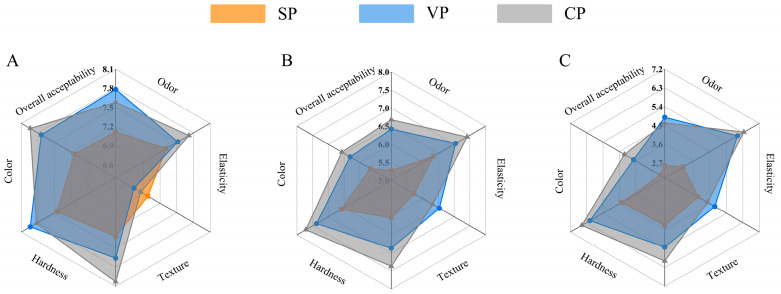
Sensory evaluation of SP, VP, and CP samples after 4 days (**A**), 6 days (**B**), and 10 days (**C**) (as representatives) of chilled storage, respectively. SP, simple packaging; VP, vacuum packing; and CP, stable chlorine dioxide pretreatment combined with vacuum packing.

A sensory evaluation is commonly used as an intuitive indicator to evaluate the freshness and quality of fish products. The results of the sensory evaluation of pike-eel samples are given in Figure 1. The sensory scores of samples all showed declining trends with increasing storage duration. Fresh pike-eel samples (0 days) were bright and shiny, with a typical fresh fish flavor (data were not provided). However, the sensory scores of the simple packaging (SP) samples decreased significantly (*p* < 0.05) after 4 days of chilled storage (Figure 1A). The notable quality deterioration was indicated in these samples by a worsening odor, changing color, and softening of muscle tissues. In contrast, the overall acceptability of the vacuum packing (VP) and stable chlorine dioxide pretreatment combined with vacuum packing (CP) samples was much better than that of the SP samples, which might be related to the absence of oxygen in the vacuum-packaging conditions. The lack of oxygen inhibits the growth of spoilage microorganisms and slows the oxidation of proteins and lipids in the muscle samples [15]. In addition, it was also found that the texture of the VP and CP samples was slightly worse than that of SP samples, which was likely attributed to the shrinkage of muscle fibers during the vacuum-packing process. This likely resulted in some drip loss from the myofiber space and, thus, a lower texture sensory score.

After 6/10 days of chilled storage (Figure 1B,C), the SP samples showed lower sensory scores than those of the other two samples. Significantly, the CP samples were in comparatively good sensory condition during the entire storage period. In contrast, the SP samples stored on day 10 had obvious spoilage odor and were rejected by the evaluation team. Overall, the CP samples showed better sensory properties than the VP and SP samples. This was mainly due to the antibacterial effects of stable chlorine dioxide during the pre-soaking process, which greatly inhibited microbial growth and metabolic actions. It also slowed the deterioration rate in the elasticity and hardness of muscle tissues. Stable chlorine dioxide was reported to have good sterilization effects against several bacteria, e.g., *Bacillus anthracis*, *Bacillus cereus*, *Clostridium perfringens*, and *Legionella* sp., as well as a better food-safety performance than gaseous chlorine dioxide [16]. In addition, vacuum packaging could effectively retard the growth of spoilage-related and pathogenic microorganisms, especially aerobic microorganisms [17]. Overall, the combination treatment resulted in the best outcomes on the muscle quality of pike-eel products during chilled storage.

### 3.2. Total Volatile Basic Nitrogen Content Analysis

**Figure 2:**
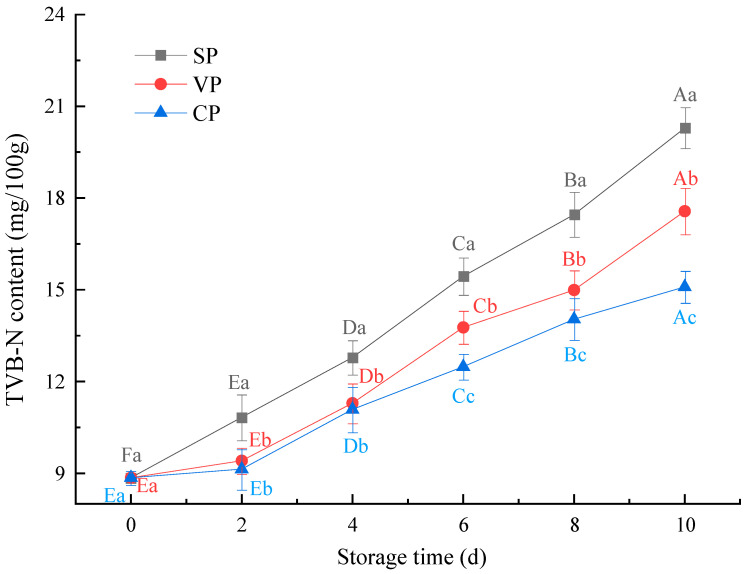
Changes in the TVB-N content of pike eels over 10 days of chilled storage. SP, simple packaging; VP, vacuum packing; and CP, stable chlorine dioxide pretreatment combined with vacuum packing. Different uppercase letters indicate significant differences in the TVB-N content of pike eels during 10 days of chilled storage (*p* < 0.05). Different lowercase letters indicate significant differences in the TVB-N content of pike eels for the same storage time (*p* < 0.05).

TVB-N includes ammonia- and amine-based compounds produced from the decomposition of muscle proteins mainly through the degradation actions of endogenous enzymes, bacterial growth, and its metabolites, and it is commonly used as an important indicator to assess the quality of fish products [18]. An increase in TVB-N content also indicates the oxidation of proteins and nonprotein substances in muscle products [16]. For the present study, the changes in TVB-N content of pike-eel samples treated with different treatments are shown in Figure 2. The initial TVB-N content was 8.85 mg N/100 g in fresh pike-eel muscle. After 10 days of storage, the TVB-N content increased gradually in all the pike-eel samples, but it did not exceed the upper limit for marine fishes (30 mg/100 g muscle) [19]. Compared to SP samples, the content of TVB-N was lower in the VP samples during the entire storage period, and this result is in agreement with a report by Genç et al. [20]. The authors found that the TVB-N content in vacuum-packaged meager (*Argyrosomus regius*) fillets was significantly lower than in samples packaged in air over 13 days of chilled storage. Additionally, the increase rate and extent of TVB-N values in the CP samples were significantly lower (*p* < 0.05) than those in the other samples during the whole storage. These results may be due to the fact that stable chlorine dioxide–pre-soaking treatments inactivated the microorganisms on the surface of pike-eel muscle samples by reacting with the proteins embedded on/in the cell membranes. Furthermore, the transmission and interaction between pike-eel muscle and air (oxygen) were physically blocked as a result of the vacuum-packaging treatment. This created anaerobic conditions that effectively prevented the growth of aerobic organisms, especially for a large number of Gram-negative bacteria [21]. Thus, the combined treatments maintained low TVB-N values in the pike-eel fillets during the chilled storage.

### 3.3. Microbiological Quality Analysis

**Figure 3:**
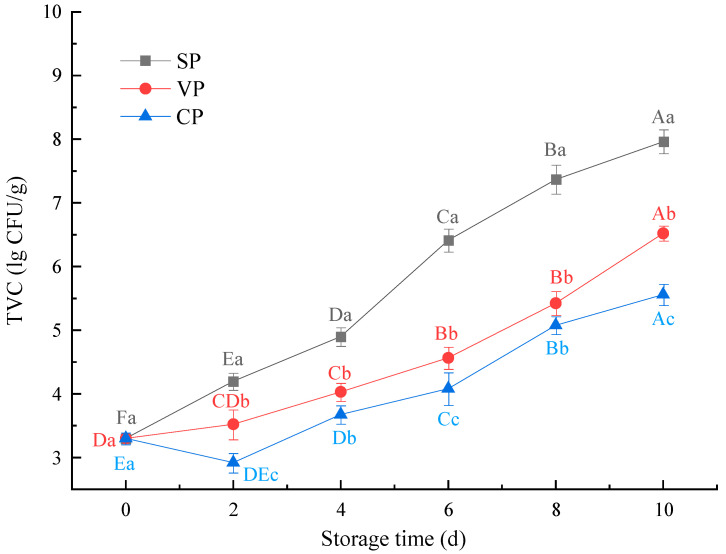
Changes in the total viable count (TVC) of pike eels during 10 days of chilled storage. SP, simple packaging; VP, vacuum packing; and CP, stable chlorine dioxide pretreatment combined with vacuum packing. Different uppercase letters indicate significant differences in the TVC of pike eels during 10 days of chilled storage (*p* < 0.05). Different lowercase letters indicate significant differences in the TVC of pike eels for the same storage time (*p* < 0.05).

The number of bacteria can reflect the spoilage degree of aquatic muscle products. Changes in the total viable count (TVC) of pike-eel samples during chilled storage are presented in Figure 3. The TVC values increased with the storage time (*p* < 0.05). After 4 days of storage, the TVC value in the SP samples (4.90 log CFU/g) was significantly higher than those in the VP (4.03 log CFU/g) and CP samples (3.68 log CFU/g). During chilled storage, the TVC values of SP samples increased more rapidly those of the VP and CP samples. According to the International Commission on Microbiological Specifications for Foods (ICMSF), a general limit of acceptance for fish is 6.60 log CFU/g [22]. A value of log 6.4 CFU/g was exceeded for samples in the SP group after 6 days of storage. On day 8, the SP group was above this limit, indicating that it had surpassed the safe consumption limit. These observations suggest the vacuum packaging and the stable chlorine dioxide pretreatments were significantly more effective than simple air packaging in terms of the inhibition of bacterial growth and multiplication. Vargas et al. reported that chlorine dioxide–soaking treatments significantly killed or inhibited spoilage microorganisms [23]. They also found chlorine dioxide treatments disrupted the outer membrane of microorganisms; this which might be the primary mechanism by which the treatment prolonged the shelf-life of pork loin under refrigerated (0–4 °C) conditions. In addition, the growth and reproduction of microorganisms can be greatly inhibited by reducing oxygen in the packaging system. Our results further validated the efficacy of stable chlorine dioxide combined with vacuum packing for the storage of chilled pike-eel products.

### 3.4. Myofibrillar Protein and Carbonyl Content Analysis

**Figure 4:**
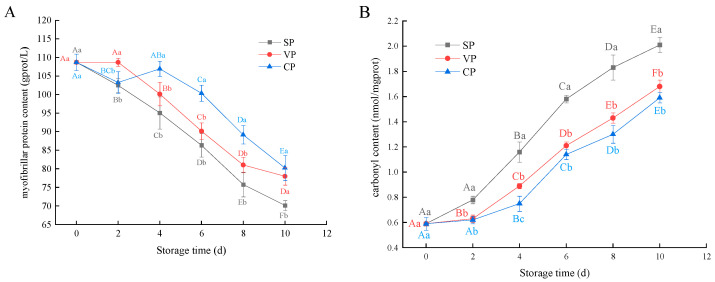
Changes in the myofibrillar protein content (**A**) and carbonyl content (**B**) of pike eels during 10 days of chilled storage. SP, simple packaging; VP, vacuum packing; and CP, stable chlorine dioxide pretreatment combined with vacuum packing. Different uppercase letters indicate significant differences in the MPs and carbonyl content of pike eels during 10 days of chilled storage (*p* < 0.05). Different lowercase letters indicate significant differences in the MPs and carbonyl content of pike eels for the same storage time (*p* < 0.05).

The physicochemical and functional properties of MPs are very important for maintaining muscle quality during storage and processing. The changes in MP content of pike-eel samples during chilled storage are shown in Figure 4A. The MP content of fresh pike eels was measured as 108.68 g of protein/L. After 10 days of storage, the MP content of the SP, VP, and CP samples was reduced significantly by 35.46%, 28.25%, and 25.26%, respectively. Previous studies also reported similar changes in MP content in peeled-shrimp (*Litopenaeus vannamei*) muscle during chilled storage [24]. The degradation/dissociation of MPs might have occurred in the pike-eel muscle during chilled storage, and the peptide bonds, disulfide bonds, and/or amino acid residues of MPs might have been destroyed or affected by the endogenous and exogenous proteases during chilled storage, resulting in significant alterations in the physicochemical and functional properties of MPs [25]. Moreover, the MPs in pike-eel muscle were degraded by specific spoilage microorganisms, which also induced considerable decreases in the MP content. Interestingly, higher MP content was found in the VP and CP samples than that in the SP samples from 4 to 10 days of storage, and this was likely due to the effective inhibition of aerobic spoilage bacteria by the vacuum-packaging treatment. The vacuum conditions facilitated the stability of MPs by inhibiting the autolysis of enzymes produced by aerobic bacterium metabolism, and the vacuum process might also promote the biochemical reaction of stable chlorine dioxide with the microorganism on the surface of pike-eel fillets [26].

The carbonyl content is commonly used to assess changes during protein oxidation due to its relatively early formation and stability [27]. The changes in the carbonyl content of pike-eel samples during chilled storage are illustrated in Figure 4B. The results indicate that the carbonyl content of all samples increased over the storage period; this result is similar to that of previous studies on the MP carbonyls determined in vacuum-packaged large yellow croaker (*Pseudosciaena crocea*) muscle during ice storage [28]. The carbonyl content of the fresh pike eels (0 days) was determined to be 0.59 nmol/mg protein. After 10 days of storage, the carbonyl content in the SP, VP, and CP samples increased to 2.01, 1.68, and 1.59 nmol/mg protein, respectively. Thus, long-term storage most likely induced the oxidation of muscle tissue [29]. The formation of carbonyls was attributed primarily to the modification of several amino acid side-chain groups, particularly those with NH-, NH_2_, or peptide-bond cleavages [30]. Additionally, the reactive oxygen species generated in tissues attacked the muscle proteins, and this increased the carbonyl content of MPs during chilled storage [31]. In the current study, the vacuum-packing treatments significantly inhibited the increase in carbonyl content in the muscle tissues. Therefore, the vacuum packing created a low-oxygen environment around the pike-eel muscle that slowed the progress of protein oxidation and inhibited the formation of carbonyl compounds.

### 3.5. Malondialdehyde Content Analysis

**Figure 5:**
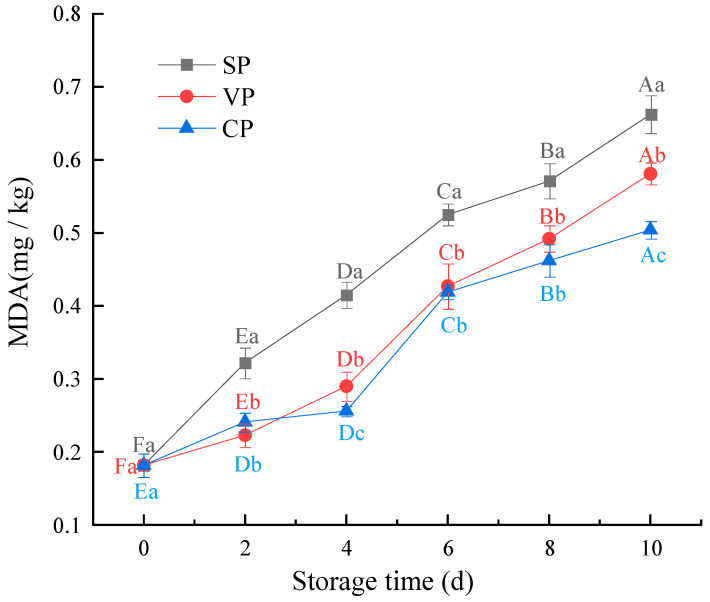
Changes in the MDA content of pike eels over 10 days of chilled storage. SP, simple packaging; VP, vacuum packing; and CP, stable chlorine dioxide pretreatment combined with vacuum packing. Different uppercase letters indicate significant differences in the MDA content of pike eels during 10 days of chilled storage (*p* < 0.05). Different lowercase letters indicate significant differences in the MDA content of pike eels for the same storage time (*p* < 0.05).

MDA is a secondary product of lipid oxidation, and it is used to evaluate the degree of lipid oxidation in animal muscle products [32]. The changes in the MDA content of pike eels during 10 days of storage can be seen in Figure 5, and the MDA content of fresh pike eels was measured as 0.182 mg/kg. After 10 days of chilled storage, the MDA content increased in all samples, and the MDA content of the VP and CP samples (0.581 mg/kg and 0.504 mg/kg, respectively) was significantly lower than in the SP samples (0.662 mg/kg) (*p* < 0.05). These changes were likely caused by the oxidation and decomposition of unsaturated fatty acids, which generated several unstable peroxides. The peroxide compounds were further converted to aldehydes, ketones, alcohols, and other shorter-chain hydrocarbons [33]. In the current study, the stable chlorine dioxide combined with vacuum-packaging treatment was more effective than the other treatments at inhibiting lipid oxidation. This was likely due to the same reasons as those mentioned for protein oxidation, as protein and lipid oxidation are closely connected in the muscle tissue. Connell suggested 1–2 mg MDA/kg of fish as the limit beyond which fish will normally develop an objectionable odor [34]. The results obtained in the present study were lower than these proposed limits, but they are similar to the results reported for *Scomber japonicus* by Zhang et al. [35].

### 3.6. Volatile Compound Analysis

**Figure 6:**
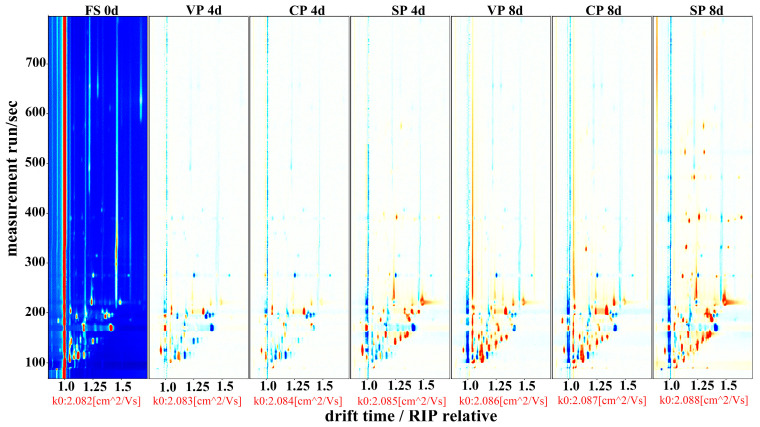
Topographical plots of volatile compounds corresponding to GC–IMS signals detected in different pike-eel samples during chilled storage. FS, fresh sample; SP, simple packaging; VP, vacuum packing; and CP, stable chlorine dioxide treatment combined with vacuum packing. Red and blue dots represent higher and lower concentrations of volatile compounds in the target samples, compared with that in the fresh samples (0 days), respectively.

GC–IMS was used to detect volatile compounds in the pike-eel samples stored on days 0 (fresh), 4, and 8 (Figure 6). The differences in the VOCs in the different samples were investigated by using the fresh sample (FS, 0 days) as the reference to deduct the original background VOCs. Two-dimensional topographic plots of volatile compounds were used to compare the similarities and differences of pike-eel samples from the three treatment groups [36]. Large numbers of VOCs were detected in FS, SP, VP, and CP samples. Compared with fresh samples, there were obvious changes in the concentration of VOCs in the SP, VP, and CP samples during chilled storage. These changes were attributed to the denaturation of proteins, oxidation of lipids/proteins, and the microbial biochemical reactions that occurred in the pike eels’ muscle tissues during chilled storage [37]. The VOCs in the SP samples increased considerably with increasing storage time, while those in the VP and CP samples did not change significantly, especially during 0–4 days of storage.

**Figure 7:**
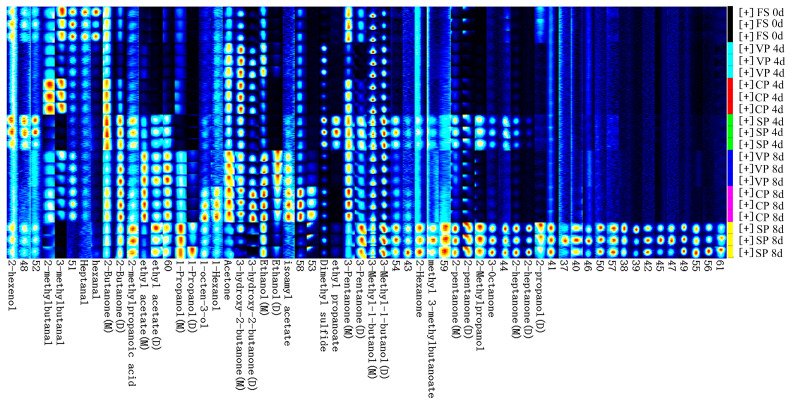
Fingerprint comparison of VOCs in the different pike-eel samples during chilled storage, as determined by GC–IMS. FS, fresh sample; SP, simple packaging; VP, vacuum packing; and CP, stable chlorine dioxide treatment combined with vacuum packing.

VOC fingerprints of the pike-eel samples were further analyzed by using FlavourSpec^®^ software. The results are presented in a gallery plot (Figure 7). A total of 61 VOCs (monomers and dimers) were detected in the pike-eel samples, including 13 ketones, 12 alcohols, 5 esters, 4 aldehydes, 1 acid, 1 thioether, and 25 unidentified volatile compounds. During chilled storage, the signal intensity of several VOCs decreased, including 2-hexenol, 2-methylbutanal, 3-methylbutanal, heptanal, and hexanal. These VOCs were also detected in the fresh samples and are likely associated with the initial fish flavor of pike-eel samples. After 4 and 8 days of chilled storage, the CP samples retained higher abundances of 2-methylbutanal and 3-methylbutanal compared with the VP and SP samples. The relatively high amount of methylbutanals might be attributed to the inhibition of muscle protein/lipid oxidation by vacuum treatments, and it is likely beneficial to the flavor quality of pike-eel samples. In addition, the signal intensity of several ketones and alcohols increased after 4 days of storage, and the number of VOCs and their signal intensities increased observably after 8 days of storage. As discussed by Dalsvåg et al., the production of ketones and alcohols is closely connected with the generation of off-flavors and odors, such as the sweet, sour, and rancid compounds formed in Atlantic salmon muscle upon chilled storage [38]. The results indicate that the VOCs of the pike eels changed significantly with prolonged chilled storage, which resulted in the deterioration of the pike-eel muscles’ flavor.

Compared with the FS samples, the abundance of 2-heptanone, 3-octanone, 2-pentanone, 2-hexanone, and 2-butanone in the SP samples also increased significantly with the increasing storage time. It was reported that ketones are mainly derived from the degradation of several amino acids, the oxidation of alcohols, and/or the decomposition of unsaturated fatty acids [39]. Ketones have relatively lower sensory threshold values, suggesting they could enhance the fishy odor through interactions with aldehydes and other small molecules [40]. In the current study, the increase in ketones in the SP samples indicates that lipid and protein oxidation likely occurred and further developed in the SP muscle tissues, thus promoting an undesirable fishy odor in the pike-eel samples. Kritikos et al. also found that the amount of propanol, isobutanol, 1-hexanol, 1-propanol, and 1-octene-3-ol in European seabass (*Dicentrarchus labrax*) fillets and Atlantic salmon (*Salmo salar*) slices increased significantly from the stage of chilled storage [41]. Additionally, 1-octen-3-ol was reported as the dominant volatile alcohol in silver carp (*Hypophthalmichthys molitrix*) [42]. Chen et al. also found that it was generated from the degradation of linoleic acid, which increased the mushroom-like and earthy odors in grass carp samples [43]. Moreover, unsaturated alcohols with relatively low threshold values had greater contributions to the volatile flavor of pike-eel samples, and they were likely derived from the degradation of fatty acid hydroperoxides and the transformation of aldehydes and ketones [39].

By comparing the abundance of VOCs across all samples, the concentration of ketones in the SP samples was much higher than that in the VP and CP samples. These findings suggest that both lipid and protein oxidation were inhibited to some extent, leading to a reduction in the VOC content in the VP and CP samples. Importantly, low levels of unsaturated alcohols were detected in the VP and CP samples on day 4. These results suggest that the degradation of fatty acids might be greatly hindered in the vacuum-packed samples during chilled storage. The abundance of 2-methylpropanoic-acid in the SP samples was higher than that in the other two groups, and it was mainly generated from the degradation of branched-chain amino acids, such as valine. These volatile organic acids commonly present characteristic odors, including rancid, sweaty, and cheese-like odors [44]. The observations further validated the effective use of both vacuum-packaging and stable chlorine dioxide treatment to inhibit the formation and development of several undesirable VOCs in the muscle samples during chilled storage.

**Figure 8:**
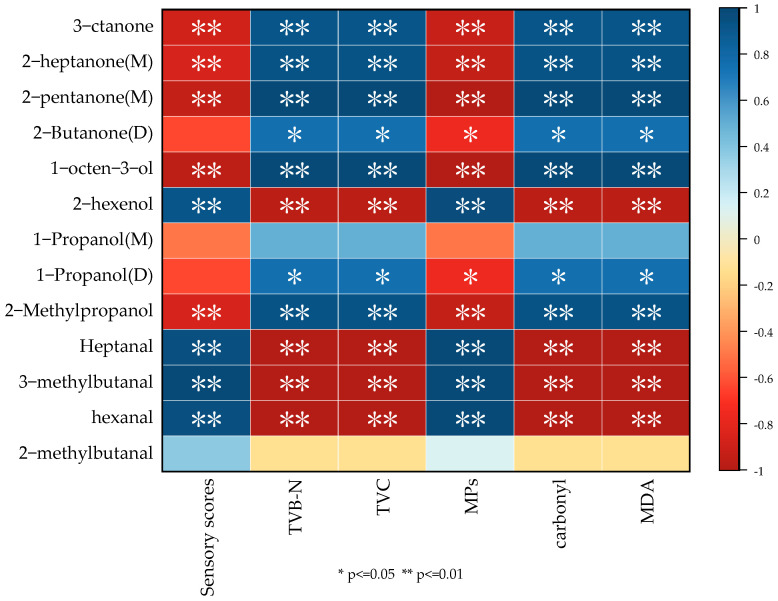
Correlation analysis between several VOCs and physicochemical properties of pike eels.

A correlation heat map was performed to determine the correlations between 13 VOCs and sensory scores, TVB-N, TVC, MP, carbonyl, and MDA content in pike eels during chilled storage by using Spearmen’s correlation tests (Figure 8). A positive correlation with remarkable difference between five ketones; three aldehydes; and the TVB-N, TVC, carbonyl, and MDA content of the samples were observed; meanwhile, these VOCs have a negative correlation between sensory scores and MP content. Additionally, no significant correlations were observed between 1-propanol(M), 2-methylbutanal, and these physicochemical parameters. This might be due to the fact that the development of lipid oxidation, the growth of bacterial, and the protein degradation led to the variations of VOCs and the deterioration of the sensory quality of pike eels during the chilled storage.

## 4. Conclusions

In this work, stable chlorine dioxide was combined with vacuum packing for the preservation of pike-eel fillets during chilled storage. The results indicate that the combined treatments significantly delayed the spoilage of pike-eel fillets during chilled storage by inhibiting bacterial growth, hindering the increase in TVB-N content, and preventing the oxidation of MPs and lipids. The results of the sensory evaluation and volatile compounds revealed that the vacuum-packaging and stable chlorine dioxide treatments significantly inhibited the development of off-flavors in the pike-eel samples. Thus, the combination of vacuum packing with stable chlorine dioxide treatment could be used as a preservation method for pike eels during chilled storage.

## Data Availability

The data presented in this study are available upon request from the corresponding author.
